# Assessment of Immobilized *Lacticaseibacillus rhamnosus* OLXAL-1 Cells on Oat Flakes for Functional Regulation of the Intestinal Microbiome in a Type-1 Diabetic Animal Model

**DOI:** 10.3390/foods13244134

**Published:** 2024-12-20

**Authors:** Grigorios Nelios, Ioanna Prapa, Gregoria Mitropoulou, Vasiliki Kompoura, Evangelos Balafas, Nikolaos Kostomitsopoulos, Amalia E. Yanni, Yiannis Kourkoutas

**Affiliations:** 1 Laboratory of Applied Microbiology and Biotechnology, Department of Molecular Biology and Genetics, Democritus University of Thrace, Dragana, 68100 Alexandroupolis, Greece; gregnelios@hotmail.com (G.N.); ioannaprap@gmail.com (I.P.); grigoriamitropoulou@gmail.com (G.M.); vickykom20.70@gmail.com (V.K.); 2 Laboratory Animal Facility, Biomedical Research Foundation of the Academy of Athens, 11527 Athens, Greece; vbalafas@bioacademy.gr (E.B.); nkostom@bioacademy.gr (N.K.); 3 Laboratory of Chemistry, Biochemistry, Physical Chemistry of Foods, Department of Nutrition and Dietetics, Harokopio University of Athens, 17671 Athens, Greece

**Keywords:** gut microbiome, presumptive probiotic, type-1 diabetes mellitus, cell immobilization, oat flakes

## Abstract

The aim of this study was to examine the effect of free or immobilized *Lacticaseibacillus rhamnosus* OLXAL-1 cells on oat flakes on the gut microbiota and metabolic and inflammatory markers in a streptozotocin (STZ)-induced Type-1 Diabetes Mellitus (T1DM) animal model. Forty-eight male Wistar rats were assigned into eight groups (n = 6): healthy or diabetic animals that received either a control diet (CD and DCD), an oat-supplemented diet (OD and DOD), a diet supplemented with free *L. rhamnosus* OLXAL-1 cells (CFC and DFC), or a diet supplemented with immobilized *L. rhamnosus* OLXAL-1 cells on oat flakes (CIC and DIC). Neither *L. rhamnosus* OLXAL-1 nor oat supplementation led to any significant positive effects on body weight, insulin levels, plasma glucose concentrations, or lipid profile parameters. *L. rhamnosus* OLXAL-1 administration resulted in a rise in the relative abundances of *Lactobacillus* and *Bifidobacterium*, as well as increased levels of lactic, acetic, and butyric acids in the feces of the diabetic animals. Additionally, supplementation with oat flakes significantly reduced the microbial populations of *E. coli*, *Enterobacteriaceae*, coliforms, staphylococci, and enterococci and lowered IL-1β levels in the blood plasma of diabetic animals. These findings suggested that probiotic food-based strategies could have a potential therapeutic role in managing dysbiosis and inflammation associated with T1DM.

## 1. Introduction

Type 1 diabetes mellitus (T1DM) is a type of chronic autoimmune disease defined by the relative or absolute lack of insulin caused by either the destruction or the dysfunction of the body’s own pancreatic beta cells [1]. T1DM has recently received universal scientific interest due to the disease’s increasing number of cases, which has been sharply rising worldwide by 3–5% per year with higher prevalence among younger demographics [2,3,4,5,6]. This alarming trend was emphasized in the International Diabetes Federation’s (IDF) 2022 report, documenting over 530,000 new cases in that year alone, 201,000 of which were among those under 20, raising the global total to approximately 8.75 million cases [7].

The mechanisms responsible for T1DM are complex, and the exact causes remain unclear since they are associated with multiple genetic predispositions and environmental factors present in those affected by the disease [8]. In this context, recent research has emphasized the role of gut microbiota as a noteworthy factor in the course of T1DM [9]. Disturbances in gut microbiome balance, also known as gut dysbiosis, have been associated with T1DM, with current evidence reinforcing a bidirectional relationship among them [10]. Specifically, it has been reported that gut dysbiosis can act as a facilitator in T1DM onset, mainly due to the initiation of autoimmune processes [11]. Meanwhile, it has also been observed that alterations in gut microbiota composition may derive as a result of the disease, aggravating the dysbiosis and potentially exacerbating diabetes symptoms for the affected individuals [12,13]. Gut dysbiosis is characterized by the imbalance of microbial communities in the intestinal tract, usually involving a decrease in beneficial bacteria, an increase in harmful bacteria, and an overall reduction of the microbial diversity within the gut environment [14]. Due to the significant impact of gut dysbiosis on T1DM, as shown by a growing number of emerging studies, restoring gut health is now considered a promising approach for the management of the disease [10,15].

In this context, probiotics are considered effective alternatives that can improve gut health by restoring the balance and function of the gut microbiome [16,17,18]. Probiotics are defined as “living microorganisms that confer beneficial effects on the host’s health when administered in adequate amounts” [19], typically recommended at a daily dose of 10⁹ CFU [20]. Despite the restoration of the gut microbiome, probiotics are shown to possess additional beneficial properties for T1DM, which can contribute to the management of the disease, such as reducing inflammation [21] and oxidative stress [22], and decreasing insulin resistance through the synthesis of short-chain fatty acids (SCFAs) [23,24].

The effectiveness of probiotics is highly dependent on their viability in the digestive tract, and cell immobilization on natural supports, a technique that confines cells within a solid natural matrix, has been shown to significantly improve cell survival after digestion, thus ensuring their health-promoting effects [25,26,27]. In a previous animal study [27], the administration of freeze-dried immobilized *Lacticaseibacillus rhamnosus* OLXAL-1 cells on oat flakes, a microbial strain studied for its antidiabetic properties [28], led to higher viability compared to freeze-dried free *L. rhamnosus* OLXAL-1 cells in mouse feces after a 10-day dietary intervention digestion protocol.

Accordingly, it is widely recognized that diet is crucial in both the management of diabetes and gut dysbiosis [29]. Integrating specific foods into an individualized diet plan can effectively address both conditions, supporting balanced blood glucose levels and promoting a healthier gut microbiome [30]. In this sense, oat flakes, known for their various nutritional and functional properties, such as reducing cholesterol levels [31], improving cardiovascular health [32,33], and diabetes management [34,35], are perceived as an excellent food carrier choice for the immobilization of probiotics and their administration to individuals with T1DM [36].

Based on these factors, the present study aimed to examine the effects of freeze-dried immobilized *L. rhamnosus* OLXAL-1 cells on oat flakes on gut microbiota modulation and blood parameters related to the disease in a streptozotocin-induced diabetic animal model, providing useful insights into potential probiotic food-based strategies for T1DM management.

## 2. Materials and Methods

### 2.1. Bacterial Strain and Growth Conditions

*Lacticaseibacillus rhamnosus* OLXAL-1, a strain isolated from olive fruits [28], was grown in a sterilized food-grade medium, consisting of 2.5% (*w*/*v*) yeast extract, 2.0% (*w*/*v*) glucose, 0.6% (*w*/*v*) CH_3_COONa, 0.2% (*w*/*v*) KH_2_PO_4_, 0.1% (*v*/*v*) Tween 80, 0.03% (*w*/*v*) MgSO_4_, and 0.005% (*w*/*v*) MnSO_4_, after incubation at 37 °C for 24 h [27,37].

### 2.2. Cell Immobilization on Oat Flakes and Preparation of Freeze-Dried Cultures

Cell immobilization and the production of freeze-dried immobilized cells on oat flakes were conducted following the methods described by Nelios et al. [27]. Initially, grown cells were collected through centrifugation (8500× *g*, 10 min, 4 °C), washed with sterile one-fourth strength Ringer’s solution (VWR International GmbH, Radnor, PA, USA), and subsequently harvested by a second centrifugation.

For cell immobilization, the harvested cells were resuspended in sterile one-fourth strength Ringer’s solution to restore the initial culture volume, forming the immobilization solution. Prior to the immobilization process, oat flakes were heat-treated at 140 °C for 30 min. The flakes were then immersed in the immobilization solution at a 30% (*w*/*v*) ratio for 15 min. After immobilization, the oat flakes were strained and rinsed with sterile one-fourth strength Ringer’s solution to remove any non-immobilized cells.

Finally, the immobilized culture was freeze-dried using a BenchTop Pro (Virtis, SP Scientific, Warminster, PA, USA) for 48 h at approximately 35 Pa, with the condenser temperature maintained at −101 °C.

### 2.3. Animals and Induction of T1DM

Male Wistar rats (RCCHan:Wistar) bred at the Laboratory Animal Facility of the Biomedical Research Foundation of the Academy of Athens (BRFAA), Greece, were used in the experiments. The animals, aged fifteen weeks, received an intraperitoneal injection of a freshly prepared streptozotocin (STZ) solution (Sigma-Aldrich (MilliporeSigma), Taufkirchen, Germany) in citrate buffer (0.1 M, pH 4.5) at a dose of 60 mg/kg. The injections were administered in the morning, and the animals were not in a fasting state. The STZ dosage was based on previous pilot studies. Six days after the STZ injection, blood glucose levels were measured, and animals with glucose concentrations exceeding 250 mg/dL [38], along with clinical signs of polyuria and polydipsia, were classified as diabetic. Glucose levels were subsequently monitored weekly to confirm the diabetic status. Animals that did not meet these criteria or failed to maintain hyperglycemia were excluded from this study. The success rate for establishing T1DM was 75%, as some animals either did not develop diabetes or succumbed to complications associated with the STZ injection. All animal procedures were reviewed and approved by the Veterinary Directorate of the Athens Prefecture (Ref. Number 272253/07-04-2021) and the Committee on Research Ethics of Democritus University of Thrace (Ref. Number 52748/559/02-06-2021) and were conducted in compliance with the European Directive 2010/63.

### 2.4. Dietary Intervention Protocol

A total of forty-eight rats were included in this study. The rats were housed individually in polysulfone Type III IVCs (Blue Line, Tecniplast, Buguggiate, Italy) with a ventilation rate of 75 air changes per h. The cages were placed in the same room, which had a HEPA-filtered air supply (15 air changes per hour), controlled temperature (21 ± 2 °C), relative humidity (55 ± 10%), and a 12:12-h light/dark cycle (light from 07:00 to 19:00). The light intensity was 300 Lux at 1 m above the floor, and the room maintained a positive air pressure of 0.6 Pa. Bedding consisted of corncob (Rehofix MK 2000, J Rettenmaier and Sons, Rosenberg, Germany), which was changed weekly. Prior to the experiment, the animals were housed individually and given a one-week acclimation period with ad libitum access to filtered tap water and standard rat chow (4RF22, Mucedola, Udine, Italy). The health of all animals in the facility was routinely monitored under a health-screening program overseen by a designated veterinarian.

The dietary intervention was conducted over a 4-week period, and the number of animals was determined based on power analysis using the “G*Power 3” software, version 3.1.9.7 and previous studies conducted by our research group [39,40,41]. The control diet consisted of standard rat chow, and all diets had similar energy content (Table 1). Rats were randomized into eight groups (4 diets × 2 health conditions) based on the dietary treatment: healthy animals that received the control diet (n = 6, CD); healthy animals that received the control diet supplemented with 2 g of freeze-dried oat flakes (10% *w*/*w* of daily diet) (n = 6, OD); healthy animals that received the control diet supplemented with 2 × 10^9^ CFU/day of freeze-dried free *L. rhamnosus* OLXAL-1 cells (n = 6, CFC); healthy animals that received the control diet supplemented with 2 × 10^9^ CFU/day of freeze-dried immobilized *L. rhamnosus* OLXAL-1 cells on oat flakes (2 g of freeze-dried immobilized culture) (n = 6, CIC); diabetic animals that received the control diet (n = 6, DCD); diabetic animals that received the control diet supplemented with 2 g of freeze-dried oat flakes (10% *w*/*w* of daily diet) (n = 6, DOD); diabetic animals that received the control diet supplemented with 2 × 10^9^ CFU/day of freeze-dried free *L. rhamnosus* OLXAL-1 cells (n = 6, DFC); diabetic animals that received the control diet supplemented with 2 × 10^9^ CFU/day of freeze-dried immobilized *L. rhamnosus* OLXAL-1 cells on oat flakes (2 g of freeze-dried immobilized culture) (n = 6, DIC).

Cell concentration of freeze-dried immobilized *L. rhamnosus* OLXAL-1 cells on oat flakes was 1 × 10^9^ CFU/g after production [27]. The daily dosage of 2 × 10^9^ CFU of either free or immobilized culture on oat flakes was based on a previous animal study [27] and typical recommendations for probiotic consumption [20]. Hence, 2 g of freeze-dried immobilized *L. rhamnosus* OLXAL-1 cells on oat flakes were administered daily to groups CIC and DIC, in order to achieve the daily dose of 2 × 10^9^ CFU. In the same manner, 2 g of freeze-dried oat flakes without cell culture were administered to the control groups. Each morning, fresh food was supplied to the animals according to their assigned diets, and any leftover chow from the previous day was discarded from the cages.

### 2.5. Sample Collection

Blood samples were obtained from the lateral tail vein at the beginning (baseline) and end (week 4) of the dietary intervention, following a six-hour fasting period. The heparinized plasma was then isolated after centrifugation (3000× *g* for 10 min at 4 °C) and stored at −80 °C until further analysis. In parallel, fresh fecal samples were collected at the same time points and stored at −20 °C. Body weight (BW) and blood glucose levels were monitored on a weekly basis using a digital glucose meter (OneTouch Verio Flex™, Lifescan Canada Ltd., Burnaby, BC, Canada) to verify the induction and maintenance of T1DM. At the conclusion of the experiment, the animals were randomly euthanized with an overdose of isoflurane (ISO-VET, Chanelle Pharma, Loughrea, Co Galway, Ireland). The hindgut segment (cecum) from all four groups was dissected, rinsed with saline, and samples of tissue and intestinal fluid were collected for microbiological analysis. Aseptic dilution of the intestinal samples (1:1) in 25% glycerol-Ringer’s solution was performed immediately, and the samples were stored at −80 °C until further analysis.

### 2.6. Determination of Metabolic and Inflammatory Markers

The concentrations of glucose, total cholesterol, triglycerides, LDL-cholesterol, HDL-cholesterol, and the liver transaminases SGOT/AST and SGPT/ALT were determined in the blood samples using an automated biochemical analyzer (Konelab 60i, Thermo Fisher Scientific, Waltham, MA, USA). In addition, insulin levels were measured using a rat insulin ELISA kit (EZRMI-13K, Merck Millipore, Darmstadt, Germany), while inflammatory markers, including tumor necrosis factor-alpha (TNF-α), interleukin-1beta (IL-1β), and interleukin-6 (IL-6), were assessed using appropriate ELISA kits (OriGene Technologies Inc., Rockville, MD, USA). Determination of inflammatory factors was performed only on blood samples collected at the end of this study, while determination of all other markers was performed in blood samples obtained at the start and the conclusion of the dietary intervention.

### 2.7. Examination of Fecal Microbial Composition

Fecal samples (~1 g) were homogenized with sterilized buffered peptone water (0.1%) (LaB M, Heywood, UK) and then diluted ten-fold using one-fourth strength Ringer’s solution. The microbiological examination involved the determination of (i) total mesophilic counts (TMC) on Plate Count Agar (PCA) (Condalab, Madrid, Spain) incubated at 30 °C for 72 h, (ii) staphylococci on Baird Parker Agar enriched with egg yolk tellurite (Condalab) at 37 °C for 48 h, (iii) coliforms on Chromogenic Coliform Agar (Condalab) at 37 °C for 24 h, (iv) *Enterobacteriaceae* on Violet Red Bile Glucose Agar (Condalab) at 37 °C for 24 h, (v) Enterococci on Kanamycin Aesculin Azide confirmatory Agar (Condalab) at 37 °C for 48 h, (vi) Lactobacilli on MRS Agar (Condalab) incubated anaerobically at 37 °C for 72 h, (vii) *Clostridium* on Tryptose Sulfite Cycloserine Agar enriched with egg yolk tellurite (Condalab), incubated anaerobically at 37 °C for 48 h, (viii) *Escherichia coli* on Tryptone Bile-X chromogenic Agar (Condalab) at 37 °C for 24 h, and (ix) Bifidobacteria on TOS Propionate Agar enriched with Lithium-Mupirocin (MUP) supplement (Condalab), incubated anaerobically at 37 °C for 48 h. Anaerobic incubation was conducted using a 2.5 L anaerobic jar (Merck Millipore) equipped with the Anaerocult A system (Merck Millipore). The duration of all incubations was extended to 120 h to confirm the accuracy of the results. The results were presented as log CFU/g of feces, considering plates with colony counts ranging from 30 to 300 per plate.

### 2.8. Analysis of Intestinal Tissue and Fluid Microbiota

Cecum tissue and cecum fluid were homogenized with sterilized buffered peptone water (0.1%) (LaB M) and then diluted ten-fold with one-fourth strength Ringer’s solution. Microbiological analyses to assess various microbial populations were carried out according to the procedures detailed in Section 2.7.

### 2.9. DNA Extraction, PCR Amplification, and 16S rRNA Sequencing

Duplicate fecal samples from each group were collected at the beginning and the 4th week of the experimental protocol for DNA extraction and Νext-Generation Sequencing (NGS). Total DNA was extracted using the NucleoSpin^®^ Stool Mini Kit (Macherey-Nagel GmbH & Co. KG, Düren, Germany) following the manufacturer’s guidelines. NGS was carried out on a MiSeq platform by MR DNA (www.mrdnalab.com, Shallowater, TX, USA), targeting the V1-V3 region of the bacterial 16S rRNA gene with 27F/519R primers (AGRGTTT-GATCMTGGCTCAG/GTNTTACNGCGGCKGCTG). PCR amplification included an initial denaturation at 94 °C for 3 min, followed by 30 cycles of 94 °C for 30 s, 53 °C for 40 s, and 72 °C for 1 min, and a final elongation step at 72 °C for 5 min. Amplified products were evaluated by electrophoresis on a 2% agarose gel, purified using Ampure XP beads (Beckman Coulter, Brea, CA, USA), and prepared for Illumina library creation according to the manufacturer’s protocol. Sequencing data were processed using MR DNA’s proprietary analysis pipeline. Operational taxonomic units (OTUs) were clustered at 3% divergence (97% similarity) and classified taxonomically using BLASTn against curated databases from RDP and NCBI (www.ncbi.nlm.nih.gov, rnacentral.org/expert-database/rdp, both accessed on 1 September 2024). Taxonomic results were compiled into “counts” and “percentage” files. OTU-level analysis and α-diversity calculations were performed using the Rhea platform [42].

### 2.10. Fecal Lactic Acid and Short Chain Fatty Acids (SCFAs) Profile

The extraction of lactic acid and SCFAs was carried out in accordance with previously established protocols [40,41]. Organic acid concentrations were assessed using an HPLC system (Shimadzu Corp., Duisburg, Germany), equipped with a Nucleogel ION 300 OA column (Macherey-Nagel, Düren, Germany), a DGU-20A5R degassing unit, an LC-20AD pump, a CTO-20AC oven at 85 °C, and a RID-10A refractive index detector. An H_2_SO_4_ solution with a concentration of 0.049 g/L was used as the mobile phase, flowing at a rate of 0.3 mL/min. The detector cell temperature was set to 60 °C. A 20 μL aliquot from each sample was injected directly into the column after filtering twice through 0.22 μm nylon filters. The concentrations of lactic acid and SCFAs were determined using standard curves with an R^2^ value of ≥0.99 [26,43].

Fecal lactic acid and SCFA concentrations were expressed as the mean μmol/g of feces, calculated using the following equation [44]:

$$SCFAs\;\&\;lactic\;acid\;(\mu mol/g)\;=\;\frac{organic\;acid\;in\;feces\;\left(\frac{mmol}{mL}\right)*Vd\left(mL\right)*1000}{weight\;of\;the\;fecal\;sample\;(g)},$$

where Vd represents the total volume of the dilution of the fecal samples.

### 2.11. Statistical Analysis

Data are presented as mean ± SD. Statistical analysis was performed using Statistica v. 12 software (StatSoft, Inc., Tulsa, OK, USA). A two-way ANOVA paired with Bonferroni post-hoc testing was applied to compare microbiota populations in intestinal tissue and fluid samples and inflammatory markers. Repeated measures ANOVA paired with Bonferroni post-hoc testing was used to evaluate microbiota populations in fecal samples, metabolic markers, and BW across the eight animal groups. Statistical significance was defined as *p* < 0.05.

## 3. Results and Discussion

### 3.1. Effect of the Dietary Intervention on Metabolic and Inflammatory Markers

**Figure 1:**
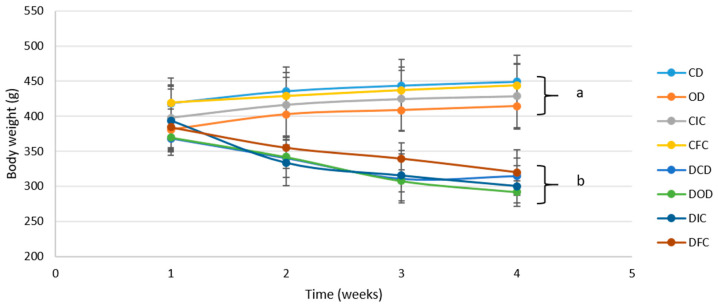
Body weight of control and diabetic rats over the 4-week dietary intervention period. The values are presented as mean ± SD (n = 6 per group). Significant differences (*p* < 0.001) among animal groups are indicated by different letters. CD: control animals following the control diet; OD: control animals following the oat flakes diet; CIC: control animals following the diet enriched with immobilized *L. rhamnosus* OLXAL-1 cells on oat flakes; CFC: control animals following the diet enriched with free *L. rhamnosus* OLXAL-1 cells; DCD: diabetic animals following the control diet; DOD: diabetic animals following the oat flakes diet; DIC: diabetic animals following the diet enriched with immobilized *L. rhamnosus* OLXAL-1 cells on oat flakes; DFC: diabetic animals following the diet enriched with free *L. rhamnosus* OLXAL-1 cells.

During the dietary intervention protocol, all diabetic animals developed symptoms characteristic of T1DM, such as polydipsia, polyuria, and polyphagia, accompanied by weight loss [45]. In particular, following 4 weeks of the dietary intervention, all diabetic groups (DCD, DOD, DIC, and DFC) exhibited a significant reduction (*p* < 0.001) in BW compared to baseline values (week 1) (Figure 1). Notably, BW was affected by the health condition (control/diabetic) from week 2 onwards and was consistently lower in the diabetic groups compared to the control groups by the end of the protocol (week 4), ranging from 291 to 319 g versus 414 to 444 g, respectively (*p* < 0.001). The observed lower BW in diabetic animals underscored the catabolic state associated with impaired glycemic control, a commonly observed phenomenon in T1DM, and evident as a consequence of STZ-induced diabetes [46,47,48]. Notably, the diets involving oat flakes or free or immobilized *L. rhamnosus* OLXAL-1 cells on oat flakes resulted in no discernible (*p* > 0.05) impact on BW.

**Figure 2:**
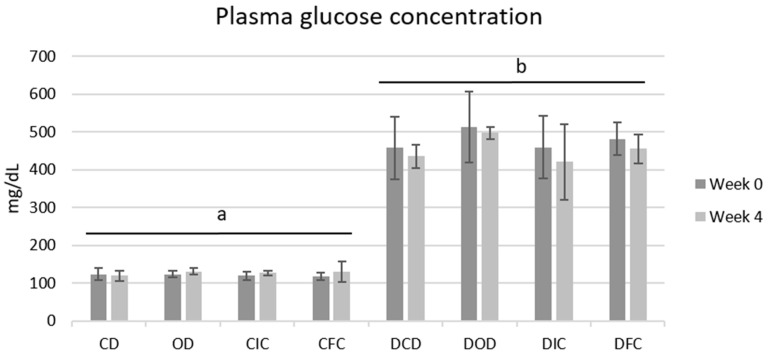
Plasma glucose concentrations of control and diabetic rats at the beginning (week 0) and the end (week 4) of the 4-week dietary protocol. The values are presented as mean ± SD (n = 6 per group). Significant differences (*p* < 0.001) among animal groups are indicated by different letters. CD: control animals following the control diet; OD: control animals following the oat flakes diet; CIC: control animals following the diet enriched with immobilized *L. rhamnosus* OLXAL-1 cells on oat flakes; CFC: control animals following the diet enriched with free *L. rhamnosus* OLXAL-1 cells; DCD: diabetic animals following the control diet; DOD: diabetic animals following the oat flakes diet; DIC: diabetic animals following the diet enriched with immobilized *L. rhamnosus* OLXAL-1 cells on oat flakes; DFC: diabetic animals following the diet enriched with free *L. rhamnosus* OLXAL-1 cells.

Corresponding to the BW, plasma glucose levels among all groups were also affected by the health condition, as expected, but not by the type of diet (Figure 2). Notably, all diabetic groups exhibited plasma glucose concentrations > 400 mg/dL with no substantial changes observed compared to their respective baseline levels (*p* = 1.000) or among various diet groups (*p* = 1.000) throughout the 4-week protocol. Similarly, plasma glucose levels of the control groups were unaffected by the different diets (*p* = 1.000), and in all cases ranged <150 mg/dL. 

**Figure 3:**
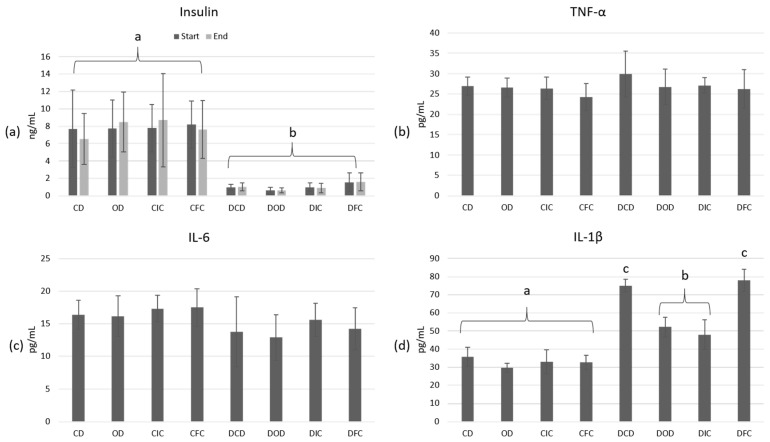
Plasma concentrations of (**a**) insulin (ng/mL) at the start and end of the dietary intervention, and (**b**) TNF-α (pg/mL), (**c**) IL-6 (pg/mL), and (**d**) IL-1β (pg/mL) at the end of the dietary intervention in control and diabetic rats. The values are presented as mean ± SD (n = 6 per group). Significant differences (*p* < 0.001) among animal groups are indicated by different letters. CD: control animals following the control diet; OD: control animals following the oat flakes diet; CIC: control animals following the diet enriched with immobilized *L. rhamnosus* OLXAL-1 cells on oat flakes; CFC: control animals following the diet enriched with free *L. rhamnosus* OLXAL-1 cells; DCD: diabetic animals following the control diet; DOD: diabetic animals following the oat flakes diet; DIC: diabetic animals following the diet enriched with immobilized *L. rhamnosus* OLXAL-1 cells on oat flakes; DFC: diabetic animals following the diet enriched with free *L. rhamnosus* OLXAL-1 cells.

At the initiation of this study (week 0), plasma insulin concentrations were significantly lower (*p* < 0.001) in the diabetic groups in comparison to the control groups (Figure 3a). Following the 4-week dietary intervention, insulin levels remained consistently low (< 2 ng/mL) across all diabetic animal groups, with no significant effect observed from the administration of oat flakes or free or immobilized *L. rhamnosus* OLXAL-1 cells (*p* > 0.05). Plasma insulin concentrations of the control groups were recorded at > 6 ng/mL in all cases and were similarly not significantly affected (*p* = 1.000) by the type of diet.

Similar studies have previously shown the impact of dietary interventions on metabolic health indicators like BW, insulin, and plasma glucose levels in T1DM animal models. For instance, Wang et al. [49] reported that the administration of all three varying doses of oat β-glucan (0.275, 0.55, and 1.1 g/kg·BW) to a male Sprague Dawley STZ-induced T1DM animal model for a period of 8 weeks led to significantly decreased plasma glucose levels. Similarly, Zhu et al. [50] found that the administration of a whole oat flour diet to male Sprague Dawley rats with STZ-induced type 2 diabetes mellitus (T2DM) for 9 weeks led to lower fasting blood glucose, higher plasma insulin levels, and reduced HbA1c levels versus the control group. Additionally, the administration of *Lactobacillus* strains has also been reported to positively affect these parameters in similar diabetic animal models [51,52].

In contrast to these studies, supplementation with either oat flakes or *L. rhamnosus* OLXAL-1 had no positive effects on the metabolic markers examined. This absence of impact from the dietary intervention on BW and plasma glucose levels could mainly be due to the dominant influence of T1DM on glycemic control or, to a lesser extent, to insufficient dosage of the bioactive compound (e.g., oat β-glucan) or the duration of the protocol. Since T1DM involves a significant decrease in insulin levels due to the destruction of insulin-producing pancreatic beta cells, dietary adjustments, though supportive of insulin therapy, cannot replace insulin’s essential role. Therefore, insulin therapy remains the primary treatment for managing blood glucose levels in T1DM [53].

To assess the impact of STZ-induced T1DM and the effects of the different diets on inflammation, the levels of TNF-α (Figure 3b), IL-6 (Figure 3c), and IL-1β (Figure 3d) in blood plasma were determined at the end of this study. According to the results, inflammation markers TNF-α and IL-6 were not affected by either the health condition (*p* = 1.000) or diet type (*p* = 1.000), and in all cases, they ranged > 20.11 pg/mL and 5.89 pg/mL, respectively. In contrast, IL-1β levels were significantly elevated in all diabetic animals compared to the control groups (*p* < 0.001). Intriguingly, a substantial reduction (*p* < 0.001) in IL-1β levels was observed in the diabetic DOD and DIC groups when compared to DCD and DFC groups, with values ranging from 48.03 to 52.23 pg/mL and 74.9 to 77.94 pg/mL, respectively, indicating a potential anti-inflammatory effect of oat flakes in diabetic animals. 

Elevated IL-1β levels are a common feature of diabetes mellitus and are directly related to many disease processes. Overexpression of IL-1β has been linked to the initiation and maintenance of a chronic low-grade inflammation in diabetic patients [54], insulin resistance [55], and the destruction of insulin-producing pancreatic beta cells [56]. Because of these observations, IL-1β has emerged as a target in the management of diabetes with examples of treatment approaches, including the use of IL-1β inhibitors, which have shown promising results in reducing inflammation and improving immune function in diabetic patients [57,58,59,60]. Oats are rich in bioactive compounds, including β-glucans and phenolic compounds, such as avenanthramides (AVAs), known for their anti-inflammatory properties [61,62,63]. In accordance with the findings of this study, Zhu et al. [50] reported that the administration of a whole oat diet to a high-fat diabetic (HFD) Sprague-Dawley animal model significantly reduced the plasma levels and mRNA expression of the proinflammatory cytokine IL-1β compared to the control HFD group. Similarly, Wang et al. [64] demonstrated that in a HFD-fed Sprague Dawley rat model, oat supplementation decreased IL-1β levels in plasma, along with reductions in other inflammatory markers, such as TNF-α and IL-6.

**Figure 4:**
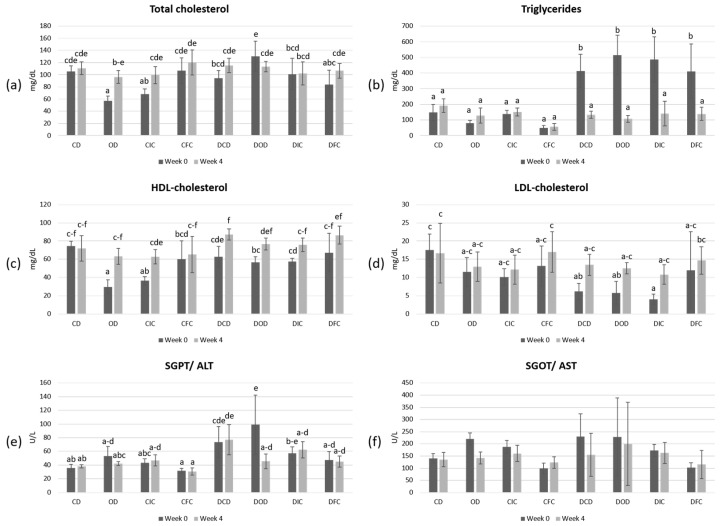
Plasma concentrations of (**a**) total cholesterol (mg/dL), (**b**) triglycerides (mg/dL), (**c**) HDL-cholesterol (mg/dL), (**d**) LDL-cholesterol (mg/dL), (**e**) SGPT/ALT (U/L), and (**f**) SGOT/AST (U/L) in control and diabetic rats at the beginning (week 0) and the end (week 4) of the 4-week dietary protocol. The values are presented as mean ± SD (n = 6 per group). Significant differences (*p* < 0.05) among animal groups are indicated by different letters. CD: control animals following the control diet; OD: control animals following the oat flakes diet; CIC: control animals following the diet enriched with immobilized *L. rhamnosus* OLXAL-1 cells on oat flakes; CFC: control animals following the diet enriched with free *L. rhamnosus* OLXAL-1 cells; DCD: diabetic animals following the control diet; DOD: diabetic animals following the oat flakes diet; DIC: diabetic animals following the diet enriched with immobilized *L. rhamnosus* OLXAL-1 cells on oat flakes; DFC: diabetic animals following the diet enriched with free *L. rhamnosus* OLXAL-1 cells.

The levels of total cholesterol, triglycerides, LDL-cholesterol, HDL-cholesterol, and the liver transaminases SGPT/ALT and SGOT/AST (Figure 4) were also determined in order to evaluate the effects of the dietary intervention in both healthy and diabetic animal models.

According to the results, levels of total cholesterol, triglycerides, HDL-cholesterol, LDL-cholesterol, and SGPT/ALT were significantly (*p* < 0.05) affected by the health condition. In addition, the diet type significantly (*p* < 0.05) affected the levels of total cholesterol, LDL-cholesterol, SGOT/ALT, and SGPT/ALT. Moreover, post-hoc analysis showed elevated (*p* < 0.05) levels of triglycerides in all diabetic groups at the beginning of the experimental protocol (week 0), which reverted to baseline levels by the end of this study (week 4).

High concentrations of triglycerides have been recognized as a contributing factor in the development of diabetes, especially in the case of T2DM, with studies showing that individuals with elevated triglycerides are more likely to experience insulin resistance, which is a critical component of diabetes pathology [65,66,67]. However, the relationship between triglycerides and T1DM seems to present a different context. In T1DM, it has been reported that poor blood glucose management can lead to elevated triglycerides due to increased lipolysis and reduced clearance of triglycerides from the bloodstream. In such cases, elevated triglyceride levels are commonly addressed through effective insulin therapy, which is essential not only for blood sugar regulation, but also for managing lipid profiles [68,69]. In our study, the normalization of triglyceride levels could be possibly attributed to improved metabolic regulation over the course of the experimental period, likely due to the natural adaptation of the animals to their diabetic condition [70]. This change seems to be independent of the diet type, as neither oat flakes nor *L. rhamnosus* OLXAL-1 supplementation had a significant impact on triglyceride levels in both control and diabetic groups by the end of the protocol.

### 3.2. Effect of the Dietary Intervention on Fecal, Intestinal Tissue, and Fluid Microbiota

**Figure 5:**
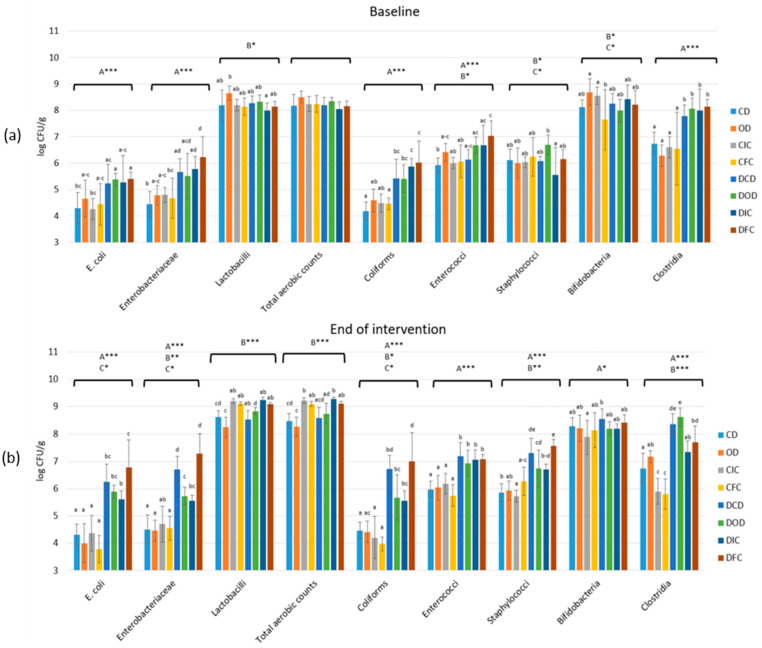
Effect of the dietary intervention on fecal microbiota populations in both control and diabetic rats at (**a**) the beginning and (**b**) the end of the 4-week protocol. The values are presented as mean ± SD (n = 6 per group). Uppercase letters A, B, and C, respectively, denote overall health condition effect, overall diet effect, and interaction between health condition and diet type. * *p* < 0.05; ** *p* < 0.01; *** *p* < 0.001. Significant differences (*p* < 0.05) among animal groups for each bacteria population within the same time point are indicated by lowercase different letters. CD: control animals following the control diet; OD: control animals following the oat flakes diet; CIC: control animals following the diet enriched with immobilized *L. rhamnosus* OLXAL-1 cells on oat flakes; CFC: control animals following the diet enriched with free *L. rhamnosus* OLXAL-1 cells; DCD: diabetic animals following the control diet; DOD: diabetic animals following the oat flakes diet; DIC: diabetic animals following the diet enriched with immobilized *L. rhamnosus* OLXAL-1 cells on oat flakes; DFC: diabetic animals following the diet enriched with free *L. rhamnosus* OLXAL-1 cells.

Microbiological examination of fecal samples (Figure 5) showed that the populations of *E. coli*, *Enterobacteriaceae*, coliforms, enterococci, staphylococci, and clostridia were overall significantly affected (*p* < 0.001) by the health condition. The type of diet affected TMC (*p* < 0.001) and the microbial populations of *Enterobacteriaceae* (*p* < 0.01), lactobacilli (*p* < 0.01), staphylococci (*p* < 0.01), and clostridia (*p* < 0.001). An interaction between the health condition and the type of diet was recorded in the populations of *Enterobacteriaceae* (*p* < 0.01) and coliforms (*p* < 0.05), while interactions between the health condition and time (baseline vs. end of intervention) were observed in TMC (*p* < 0.05) and the populations of *E. coli* (*p* < 0.001), *Enterobacteriaceae* (*p* < 0.001), lactobacilli (*p* < 0.05), coliforms (*p* < 0.001), enterococci (*p* < 0.01), and staphylococci (*p* < 0.001). An interaction between the type of diet and time was observed in TMC (*p* < 0.001) and the populations of *E. coli* (*p* < 0.05), *Enterobacteriaceae* (*p* < 0.05), lactobacilli *(p* < 0.001), coliforms (*p* < 0.01), enterococci (*p* < 0.05), and staphylococci (*p* < 0.05). Finally, interactions among the health condition, the type of diet, and time were detected in the populations of lactobacilli (*p* < 0.01) and staphylococci (*p* < 0.05).

Baseline levels of *E. coli*, *Enterobacteriaceae*, coliforms, and clostridia in fecal samples of all diabetic groups (DCD, DOD, DIC, and DFC) were significantly higher (*p* < 0.001) compared to the control groups (CD, OD, CIC, and CFC). Additionally, by the end of the dietary intervention protocol, levels of *E. coli*, *Enterobacteriaceae*, coliforms, and clostridia were further increased (*p* < 0.05) in the feces of diabetic groups compared to baseline levels. Increased levels (*p* < 0.05) of staphylococci and enterococci were also determined in the feces of diabetic groups that were collected at the conclusion of the dietary intervention, compared to baseline levels. Remarkably, despite being higher than the initial levels, the fecal samples from diabetic DOD and DIC groups exhibited significantly lower levels (*p* < 0.05) of *E. coli*, *Enterobacteriaceae*, coliforms, and staphylococci compared to DCD and DFC groups. Finally, a significant increase (*p* < 0.05) in lactobacilli populations in the fecal samples collected by the end of the intervention in the control CIC and CFC groups, as well as in the diabetic DIC and DFC groups, was observed compared to both baseline levels and the groups that did not receive *L. rhamnosus* OLXAL-1 cells (week 4).

Microbiological analysis of the intestinal tissue and the fluid samples was conducted to further assess the dietary intervention’s effects on gut microbiota, with the cecum selected as the target of the analysis because of its significant microbial diversity and abundance [71,72]. According to the results (Table 2), the health condition affected the populations of *E. coli* (*p* < 0.001), *Enterobacteriaceae* (*p* < 0.001), coliforms (*p* < 0.001), enterococci (*p* < 0.001), staphylococci (*p* < 0.001), and clostridia (*p* < 0.001) in both the cecum tissue and the fluid samples. The type of the diet also exhibited a significant (*p* < 0.001) effect on all the studied bacterial populations in the cecum fluid samples, with similar results observed for the cecum tissue samples (*p* < 0.01). Significant interactions were observed between the health condition and the type of diet, in both the cecum tissue and the fluid samples, concerning the populations of bifidobacteria (*p* < 0.01) and clostridia (*p* < 0.001).

Based on the results of the microbiological analyses, populations of *E. coli, Enterobacteriaceae*, coliforms, enterococci, staphylococci, and clostridia were significantly affected by the onset of STZ-induced T1DM. This initial increase in the above bacterial populations in the fecal samples of diabetic animals, observed at the beginning of the experimental protocol, has been previously reported and can be seen as a sign of dysbiosis in the gut microbiota associated with the disease [40,41]. Furthermore, the continued increase in the already elevated populations of these potentially pathogenic bacteria only in diabetic animals emphasizes the progressive impact of T1DM on the development of gut dysbiosis over time.

Moreover, the type of diet also played a crucial role in affecting microbial populations, such as *Enterobacteriaceae,* coliforms, and lactobacilli, indicating that dietary components can modulate gut microbiota composition significantly [73]. Interestingly, while the diabetic groups exhibited higher baseline levels of potentially pathogenic bacteria compared to the healthy groups, those receiving oat supplementation (groups DOD and DIC) had significantly lower levels of *E. coli*, *Enterobacteriaceae*, coliforms, and staphylococci compared to the rest of the diabetic groups on oat-free diets (groups DCD and DFC), suggesting that oat flake supplementation may play a beneficial role in mitigating dysbiosis in diabetic rats. Dietary fibers, particularly those found in oats like β-glucans, are known for their function as prebiotics since they are recognized for stimulating the growth of beneficial microbial populations, which can help suppress the proliferation of harmful bacteria [74]. This aligns with findings from various studies, indicating that whole grains and their components can significantly alter gut microbiota profiles, enhancing microbial diversity and stability [50].

### 3.3. Effect of the Dietary Intervention on the Fecal Microbiome Using Next-Generation Sequencing

Fecal samples of healthy and diabetic animals were further investigated using Next-Generation Sequencing (NGS) to examine the impact of the dietary intervention on the composition and diversity of the intestinal microbiome.

α-diversity is a critical parameter in microbiome studies, as it refers to the different microbial populations and includes the number of different species and the balance between them in the sample under analysis. As diet influences the composition of the microbiome, α-diversity can act as a sensitive indicator to assess the microbiome’s response to dietary interventions. The Simpson and Shannon indices are indicators used to determine α-diversity, which, considering the abundance and equal distribution of different microbial species in the sample, provide information on the complexity and homogeneity of the microbiome. The interpretation of these two indices is inverse, with a low value of the Shannon index indicating low diversity of the sample, while a low value of the Simpson index indicates high diversity [75].

**Figure 6:**
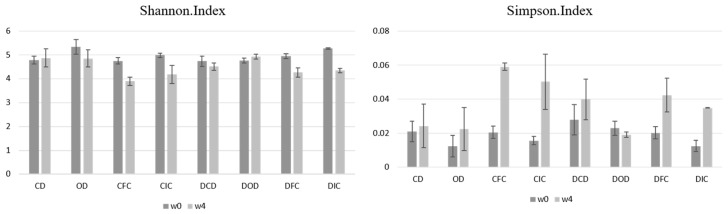
Effect of the dietary intervention on Shannon and Simpson index values in fecal samples of healthy and diabetic rats, as determined by 16S rRNA NGS at the beginning (w0) and the end (w4) of the 4-week protocol. CD: control animals following the control diet; OD: control animals following the oat flakes diet; CIC: control animals following the diet enriched with immobilized *L. rhamnosus* OLXAL-1 cells on oat flakes; CFC: control animals following the diet enriched with free *L. rhamnosus* OLXAL-1 cells; DCD: diabetic animals following the control diet; DOD: diabetic animals following the oat flakes diet; DIC: diabetic animals following the diet enriched with immobilized *L. rhamnosus* OLXAL-1 cells on oat flakes; DFC: diabetic animals following the diet enriched with free *L. rhamnosus* OLXAL-1 cells.

According to the results (Figure 6), although changes were observed in both indices, they were not significant (*p* > 0.05). Specifically, neither index was affected by the health status (*p* > 0.05), the diet type (*p* > 0.05), or their interaction (*p* > 0.05).

At the phylum level (Table 3), Firmicutes and Bacteroidetes were the most prevalent in the feces of all groups studied, with relative abundances ranging from 60.00% to 90.00% for Firmicutes and 4.10% to 39.21% for Bacteroidetes. The health condition and the diet type significantly affected the relative abundances of Actinobacteria (*p* < 0.001), as a significant increase (*p* < 0.001) was observed in the feces of the DFC group after the conclusion of the dietary intervention (11.21%) versus the initial levels (0.71%), whereas no other significant variation was observed for the other phyla.

**Figure 7:**
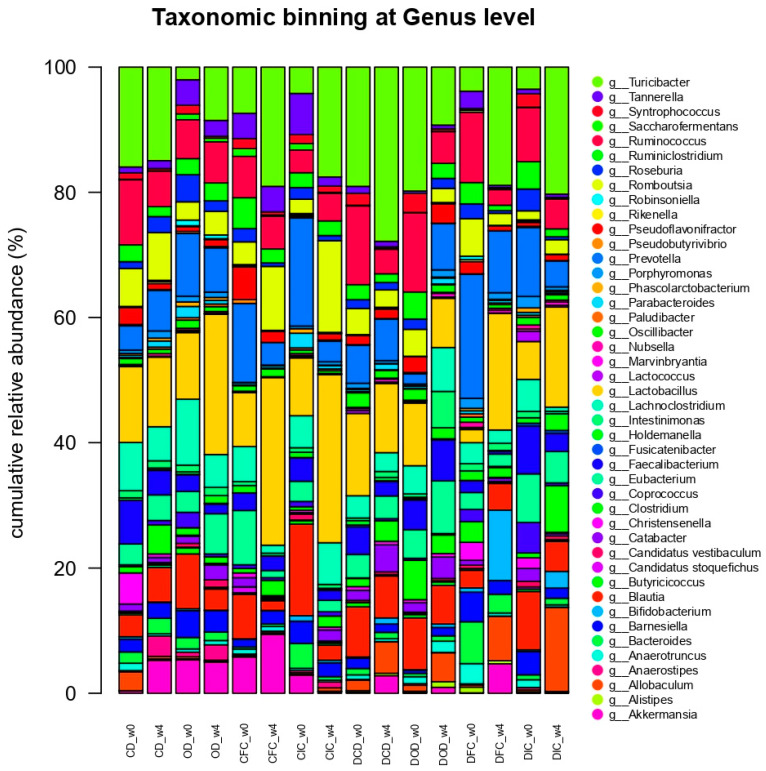
Effect of the dietary intervention on the genus-level taxonomic binning with normalized relative abundances (%) in the feces of control and diabetic rats, as determined through 16S rRNA NGS at the beginning (w0) and the end (w4) of the 4-week protocol (n = 2 per group). CD: control animals following the control diet; OD: control animals following the oat flakes diet; CIC: control animals following the diet enriched with immobilized *L. rhamnosus* OLXAL-1 cells on oat flakes; CFC: control animals following the diet enriched with free *L. rhamnosus* OLXAL-1 cells; DCD: diabetic animals following the control diet; DOD: diabetic animals following the oat flakes diet; DIC: diabetic animals following the diet enriched with immobilized *L. rhamnosus* OLXAL-1 cells on oat flakes; DFC: diabetic animals following the diet enriched with free *L. rhamnosus* OLXAL-1 cells.

At the genus level (Figure 7), statistical analysis demonstrated significant changes in the relative abundances of the genera *Lactobacillus* and *Bifidobacterium*. For the *Lactobacillus* genus, both the health condition (*p* < 0.05) and the diet type (*p* < 0.001) had a significant effect, with a notable interaction between the health condition and the diet type (*p* < 0.05). Specifically, increased (*p* < 0.05) relative abundances of *Lactobacillus* were determined in the feces of the healthy CIC and CFC groups compared to the CD and OD groups after the end of the experimental protocol. Similarly, increased levels of *Lactobacillus* were identified in the DIC and DFC groups, compared to the DCD and DOD groups; however, this increase was not significant (*p* > 0.05).

Likewise, relative abundances of *Bifidobacterium* were significantly influenced by the health condition (*p* < 0.001) and the diet type (*p* < 0.001), as increased percentages of *Bifidobacterium* were recorded in the feces of the DFC group, collected at the end of the experimental protocol (week 4), compared to baseline (week 0) and the rest of the groups for the same time point (week 4).

In general, similar studies exploring changes in gut microbiome in metabolic diseases like T1DM and T2DM often focus on shifts in the Firmicutes/Bacteroidetes ratio. For instance, in a study where diabetes was induced in mice through STZ administration, a rise in the Firmicutes/Bacteroidetes ratio was reported in the diabetic models compared to the healthy controls [76,77]. In contrast, a similar study that investigated changes in the gut microbiome of a Sprague-Dawley diabetic animal model following STZ-induced T1DM reported a decrease in the Firmicutes/Bacteroidetes ratio in the diabetic models versus the controls [75]. Moreover, clinical studies examining changes in the microbiome of adults and juveniles with T1DM observed a decrease in the Firmicutes/Bacteroidetes ratio [16,78]. Finally, some cases have also reported no significant change in this ratio between diabetic patients and healthy subjects [79], an observation consistent with the outcomes of this study.

In the same manner, many studies have documented a connection between reduced microbiome diversity and intestinal dysbiosis in metabolic diseases, such as T1DM [80]. Additionally, numerous studies have also reported a link between increased diversity and positive health outcomes [81]. However, the literature on α-diversity markers in diabetic animal models is also highly controversial, with values varying based on factors, such as gender, age, species, and other experimental parameters [82].

Moreover, the changes observed in our study at the genus level, particularly the increase in the relative abundance of the *Lactobacillus* genus in the feces of both healthy and diabetic groups receiving *L. rhamnosus* OLXAL-1 cells, align with the results of the microbiological analyses, suggesting that *L. rhamnosus* OLXAL-1 cells are capable of surviving through the gastrointestinal tract. In addition, the increase in the relative abundance of the *Bifidobacterium* genus in the feces of the diabetic group receiving free *L. rhamnosus* OLXAL-1 culture (DFC group) aligns with the results of the study by Wang et al. [76], where the administration of *L. rhamnosus* YC to diabetic C57BL/6J mice for 5 weeks resulted in a similar rise in the relative abundance of this genus.

Finally, it is worth noting that in the present study, no significant differences were observed in the abundance of the *Akkermansia* genus. *Akkermansia muciniphila* is considered a next-generation probiotic microorganism that has been correlated with the progression of autoimmune T1DM. Specifically, according to the influential study by Hänninen et al. [83], treatment with *A. muciniphila* delayed the onset of autoimmune T1DM in a non-obese diabetic (NOD) mouse model, suggesting its protective effect against the development of the disease through immunomodulatory properties and maintenance of gut homeostasis.

### 3.4. Effect of the Dietary Intervention on Fecal Lactic Acid and Short Chain Fatty Acids (SCFAs) Profile

Several studies in T1DM have shown that the production of SCFAs may have positive health effects on the symptoms of the disease. SCFAs can contribute to the proliferation of intestinal epithelial cells, enhancing the intestinal barrier function [84], activating receptors in the pancreas and liver, improving glycolipid metabolism [85], and reducing insulin resistance and inflammation levels in T1DM by interacting with free fatty acid receptors [86]. Based on these factors, the levels of fecal lactic acid and SCFAs of both control and diabetic rats were determined to evaluate the impact of T1DM and different diets on gut metabolic activity (Table 4).

Overall, during the 4-week experimental protocol, significant alterations in the levels of lactic acid and SCFAs in the feces of the animals were determined. In particular, lactic acid concentration was significantly affected by the health condition (*p* < 0.001) and the diet type (*p* < 0.001), but no strong interactions were observed between these two factors. Acetic acid concentration was significantly influenced by the health condition (*p* < 0.001), the diet type (*p* < 0.001), and a strong (*p* < 0.001) interaction between them, as well as between the health condition and the time (*p* < 0.001) were observed. Similarly, propionic acid concentration was affected by the health condition (*p* < 0.001) and the diet type (*p* < 0.001). Finally, isobutyric, butyric, and valeric acid concentrations were significantly influenced by the health condition, the diet type, and the time (*p* < 0.001, in all cases).

In addition, further post-hoc analysis revealed several key findings among the animal groups studied. Notably, a significant increase (*p* < 0.001) in lactic acid concentration was observed in the feces of both healthy and diabetic groups that received either free or immobilized *L. rhamnosus* OLXAL-1 cells, compared to groups without *L. rhamnosus* OLXAL-1 supplementation. Importantly, the administration of immobilized *L. rhamnosus* OLXAL-1 cells on oat flakes led to a significant increase (*p* < 0.001) in butyric acid levels in the feces of the CIC group compared to all other groups. Finally, acetic acid levels, which were already elevated (*p* < 0.05) in the feces of all diabetic rats at the beginning of the protocol compared to the control groups (week 0), were further increased (*p* < 0.001) in the feces of DFC and DIC groups versus CD and DOD groups.

Alterations in SCFAs levels have been previously linked with the regulation of the gut flora by probiotic microorganisms [87], with butyric, acetic, and propionic acids being the most studied and considered the most important compounds for the host health [88,89]. According to the results of our study, an increase in acetic acid levels was observed in diabetic animal models receiving *L. rhamnosus* OLXAL-1 culture. In a previous study, the administration of acetic acid to a high-fat diet C57BL/6 mouse model resulted in the control of food appetite and a reduction in adipose tissue accumulation [90]. In addition, the administration of immobilized *L. rhamnosus* OLXAL-1 cells on oat flakes resulted in a significant increase in butyric acid in the feces of the control animals. Previous research has shown that the addition of butyric acid to the diet can prevent the development of insulin resistance and obesity in animal models following a high-fat diet [91]. Additionally, it has been demonstrated that butyric acid administration can lead to a decrease in blood glucose levels and an increase in insulin levels in animal models with induced diabetes after STZ administration [92]. Finally, the administration of *L. rhamnosus* OLXAL-1 cells also led to a significant rise in lactic acid levels in the feces of both healthy and diabetic animals, which may facilitate the management of symptoms of metabolic diseases, such as T1DM, as the administration of this acid has been associated with antimicrobial and immunomodulatory properties [93].

### 3.5. Study’s Limitations and Future Perspectives

While this study provides valuable findings, several factors should be considered when interpreting the results.

Firstly, the use of an STZ-induced model may not fully reflect the characteristics of naturally occurring T1DM in humans, which could influence the applicability of the results. As previously mentioned, naturally occurring T1DM is characterized by the gradual destruction of pancreatic beta cells by the host’s immune system, ultimately leading to the lack of insulin. In contrast, STZ-induced diabetes is caused by the direct cytotoxic effects of STZ on beta cells [38], a method that bypasses the autoimmune processes involved in the onset of T1DM, resulting thus in differences in disease progression, immune responses, and associated conditions [94].

Another point to consider is the sample size in the NGS analysis, with only two animals per group, which may not fully capture the diversity of microbial responses and interactions. However, previous studies with the same sample size have revealed significant differences, highlighting the robustness of the observed effects [40,41,95]. For instance, the administration of *Pediococcus acidilactici* ORE5 [41] and *Pediococcus acidilactici* SK [95] cells in the same established T1DM animal model resulted in a significant increase in the abundance of the *Pediococcus* genus compared to initial values and control diets in both studies.

Looking ahead, the mechanisms of action by which oat and *L. rhamnosus* OLXAL-1 supplementation led to the observed changes require further investigation into the specific pathways involved. For example, exploring how the synergistic combination of oats and probiotics modulates the production of microbiota-derived SCFAs, such as butyric and acetic acids, in in vitro simulation models (e.g., SHIME^®^ [96]) could provide valuable insights into gut health. Future studies should also examine how the components of the current dietary intervention can modulate inflammation, particularly through the NF-κB pathway, which controls the production of pro-inflammatory cytokines, such as IL-1β [97]. Moreover, the interaction between oat fibers, probiotics, and gut microbiota in regulating gut hormones, such as leptin and ghrelin, which influence appetite and energy metabolism, could also provide significant insights [98,99]. Finally, the potential effects on the expression and regulation of enzymes involved in glucolipid metabolism, like hexokinases (HKs), glucose-6-phosphate dehydrogenase (G6PD), and acetyl-CoA carboxylase (ACC), could provide further information into the potential metabolic benefits of these interventions.

Additionally, expanding this study to include animals receiving concurrent insulin supplementation would provide significant information into how these interventions affect key metabolic markers related to insulin sensitivity and glucose homeostasis. In addition to the disease markers examined in our study, the inclusion of analyses for C-peptide levels, glycosylated hemoglobin (HbA1c), additional inflammatory cytokines (e.g., IL-4, IL-10), oxidative stress markers (e.g., catalase, superoxide dismutase—SOD, reactive oxygen species—ROS), and gut barrier integrity proteins (e.g., ZO-1, occludin, claudin) could offer a deeper understanding of the effect of the dietary intervention on the progression of the disease.

Finally, to evaluate the broader relevance of these findings, clinical trials involving human subjects with T1DM are imperative. By testing parameters like HbA1c, fasting glucose, insulin requirements, gut microbiota diversity, and quality of life assessments, such trials would be crucial for validating whether the effects reported in animal models can be replicated in humans, ultimately leading to the formulation of novel probiotic food-based strategies for T1DM management.

## 4. Conclusions

The aim of this study was to assess the effect of freeze-dried immobilized *L. rhamnosus* OLXAL-1 cells on oat flakes on blood parameters and gut microbiota modulation associated with the disease in an STZ-induced T1DM animal model. 

Prior to the dietary intervention, elevated plasma glucose and triglyceride levels, reduced plasma insulin concentrations, and higher baseline levels of microbial populations, such as *E. coli*, *Enterobacteriaceae*, coliforms, and clostridia, were observed in diabetic animals with STZ-induced T1DM compared to the healthy groups. The dietary intervention had no impact on metabolic markers like BW or plasma glucose levels, probably due to the dominant influence of T1DM. Administration of *L. rhamnosus* OLXAL-1 led to increased relative abundances of *Lactobacillus* and *Bifidobacterium*, along with higher levels of lactic, acetic, and butyric acids in the feces of diabetic animals. Additionally, oat flakes supplementation significantly decreased the populations of *E. coli*, *Enterobacteriaceae*, coliforms, staphylococci, and enterococci, while also reduced IL-1β levels in the blood plasma of diabetic animals, suggesting that oat flakes supplementation may play a beneficial role in mitigating dysbiosis that occurs in diabetes.

However, despite the above findings, further research, including well-designed clinical trials, is necessary to fully assess the therapeutic potential of such dietary interventions for the management of T1DM in humans.

## Figures and Tables

**Table 1:** Nutritional composition of the diets supplied to the animals (g/100 g).

	Control Diet	Oat Flakes Supplemented Diet
Proteins (g)	19.50	18.95
Carbohydrates (g)	41.51	43.05
Fats (g)	6.00	6.09
Dietary fiber (g)	4.20	4.73
Moisture content (%)	12.00	11.89
Ash (%)	6.00	5.48
Freeze-dried oat flakes (g)	0	10.00
Energy (Kcal)	298	302

**Table 2:** Effect of the dietary intervention on the gut microbiota populations (log CFU/g) in the cecum intestinal tissue and intestinal fluid in both control and diabetic rats.

	CD	OD	CIC	CFC	DCD	DOD	DIC	DFC
Intestinal fluid (cecum)	Cell populations (log CFU/g)							
*E. coli*	4.65 ± 0.66 ^a–c^	4.22 ± 0.69 ^a^	4.22 ± 0.69 ^a^	4.55 ± 0.35 ^ab^	6.16 ± 0.24 ^de^	5.51 ± 0.75 ^cd^	5.35 ± 0.39 ^b–d^	6.47 ± 0.28 ^e^
*Enterobacteriaceae*	4.59 ± 0.69 ^a–c^	4.08 ± 0.91 ^a^	4.35 ± 0.48 ^ab^	4.61 ± 0.38 ^a–c^	6.23 ± 0.17 ^cd^	5.42 ± 1.00 ^de^	5.31 ± 0.37 ^b–d^	6.50 ± 0.23 ^e^
Lactobacilli	7.38 ± 0.32 ^a^	7.33 ± 0.64 ^a^	8.28 ± 0.29 ^b^	8.32 ± 0.25 ^b^	7.55 ± 0.28 ^a^	7.48 ± 0.70 ^a^	8.42 ± 0.21 ^b^	8.36 ± 0.24 ^b^
Total mesophilic counts	7.40 ± 0.27 ^a^	7.40 ± 0.41 ^a^	8.29 ± 0.21 ^b^	8.27 ± 0.37 ^b^	7.56 ± 0.31 ^a^	7.41 ± 0.45 ^a^	8.44 ± 0.18 ^b^	8.31 ± 0.31 ^b^
Coliforms	4.63 ± 0.65 ^ab^	4.26 ± 0.68 ^a^	4.15 ± 0.85 ^a^	4.29 ± 0.50 ^a^	6.21 ± 0.13 ^cd^	5.43 ± 0.74 ^b–d^	5.34 ± 0.35 ^bc^	6.41 ± 0.33 ^d^
Enterococci	5.13 ± 0.12 ^cd^	4.74 ± 0.33 ^c^	5.54 ± 0.81 ^acd^	5.65 ± 0.12 ^ad^	6.32 ± 0.22 ^ab^	6.51 ± 0.36 ^b^	6.30 ± 0.49 ^ab^	6.21 ± 0.30 ^ab^
Staphylococci	5.21 ± 0.14 ^bc^	4.76 ± 0.37 ^b^	4.64 ± 0.31 ^b^	5.52 ± 0.30 ^ac^	6.24 ± 0.23 ^ad^	6.28 ± 0.41 ^d^	5.57 ± 0.65 ^ac^	6.18 ± 0.31 ^ad^
Bifidobacteria	7.27 ± 0.14 ^ab^	7.08 ± 0.08 ^ab^	7.54 ± 0.45 ^ac^	7.58 ± 0.41 ^ac^	6.57 ± 0.35 ^b^	6.74 ± 0.09 ^ab^	8.40 ± 0.27 ^c^	7.38 ± 0.78 ^ab^
Clostridia	6.00 ± 0.12 ^bc^	5.81 ± 0.16 ^b^	5.29 ± 0.32 ^e^	6.71 ± 0.42 ^a^	7.46 ± 0.20 ^d^	7.31 ± 0.17 ^d^	6.62 ± 0.26 ^ac^	6.73 ± 0.29 ^a^
Intestinal tissue (cecum)								
*E. coli*	3.43 ± 0.71 ^ab^	3.26 ± 0.67 ^a^	3.29 ± 0.76 ^a^	3.53 ± 0.44 ^ab^	5.49 ± 0.30 ^cd^	4.61 ± 0.74 ^cd^	4.36 ± 0.34 ^bc^	5.53 ± 0.36 ^d^
*Enterobacteriaceae*	3.15 ± 0.63 ^ab^	3.12 ± 0.77 ^b^	3.75 ± 0.89 ^ab^	3.76 ± 0.42 ^ab^	5.50 ± 0.21 ^cd^	4.46 ± 0.84 ^ac^	4.37 ± 0.40 ^ac^	5.55 ± 0.30 ^d^
Lactobacilli	6.04 ± 0.34 ^a^	5.99 ± 0.57 ^a^	6.94 ± 0.41 ^b^	6.97 ± 0.35 ^b^	5.86 ± 0.41 ^a^	6.11 ± 0.62 ^a^	6.87 ± 0.39 ^b^	7.05 ± 0.26 ^b^
Total aerobic counts	6.12 ± 0.31 ^a^	6.12 ± 0.33 ^a^	7.00 ± 0.30 ^b^	6.99 ± 0.39 ^b^	5.96 ± 0.19 ^a^	6.13 ± 0.37 ^a^	6.98 ± 0.20 ^b^	7.15 ± 0.21 ^b^
Coliforms	3.49 ± 0.76 ^ac^	3.12 ± 0.63 ^a^	3.01 ± 0.92 ^a^	3.16 ± 0.56 ^a^	5.45 ± 0.17 ^c^	4.57 ± 0.69 ^bc^	4.49 ± 0.39 ^bc^	5.57 ± 0.44 ^c^
Enterococci	3.84 ± 0.19 ^bc^	3.39 ± 0.28 ^b^	4.20 ± 0.77 ^bc^	4.30 ± 0.22 ^c^	5.42 ± 0.32 ^a^	5.92 ± 0.31 ^a^	5.72 ± 0.53 ^a^	5.63 ± 0.42 ^a^
Staphylococci	4.13 ± 0.17 ^bc^	3.67 ± 0.38 ^bc^	3.55 ± 0.37 ^b^	4.43 ± 0.41 ^cd^	5.76 ± 0.30 ^a^	5.84 ± 0.42 ^a^	5.14 ± 0.65 ^ad^	5.75 ± 0.42 ^a^
Bifidobacteria	5.88 ± 0.20 ^a^	5.69 ± 0.15 ^a^	6.14 ± 0.54 ^a^	6.18 ± 0.48 ^a^	5.67 ± 0.42 ^a^	5.66 ± 0.16 ^a^	7.34 ± 0.36 ^b^	6.32 ± 0.82 ^ab^
Clostridia	4.91 ± 0.26 ^ab^	4.73 ± 0.13 ^af^	4.20 ± 0.36 ^f^	5.62 ± 0.48 ^c–e^	6.36 ± 0.16 ^e^	6.03 ± 0.15 ^de^	5.16 ± 0.23 ^a–c^	5.47 ± 0.36 ^b–d^

The values are presented as mean ± SEM (n = 6 per group). Significant differences (*p* < 0.05) among animal groups, for each bacteria population, are indicated by different letters. CD: control animals following the control diet; OD: control animals following the oat flakes diet; CIC: control animals following the diet enriched with immobilized *L. rhamnosus* OLXAL-1 cells on oat flakes; CFC: control animals following the diet enriched with free *L. rhamnosus* OLXAL-1 cells; DCD: diabetic animals following the control diet; DOD: diabetic animals following the oat flakes diet; DIC: diabetic animals following the diet enriched with immobilized *L. rhamnosus* OLXAL-1 cells on oat flakes; DFC: diabetic animals following the diet enriched with free *L. rhamnosus* OLXAL-1 cells.

**Table 3:** Effect of the dietary intervention on the phylum-level taxonomic binning with normalized relative abundances (%) in rat feces, as determined by 16S rRNA NGS at the beginning (week 0) and the end (week 4) of the 4-week protocol (n = 2 per group).

Phylum	Relative Abundances (%)															
	CD		OD		CIC		CFC		DCD		DOD		DIC		DFC	
	Week 0	Week 4	Week 0	Week 4	Week 0	Week 4	Week 0	Week 4	Week 0	Week 4	Week 0	Week 4	Week 0	Week 4	Week 0	Week 4
Firmicutes	88.93 ± 3.09 ^ab^	78.22 ± 5.87 ^ab^	69.76 ± 1.07 ^ab^	76.95 ± 1.30 ^ab^	60.36 ± 7.09 ^a^	90.00 ± 8.82 ^ab^	75.01 ± 6.38 ^ab^	79.38 ± 11.19 ^ab^	87.61 ± 7.88 ^ab^	83.63 ± 12.44 ^ab^	95.67 ± 0.28 ^b^	84.46 ± 2.51 ^ab^	78.88 ± 12.85 ^ab^	87.71 ± 0.16 ^ab^	60.00 ± 8.80 ^a^	65.75 ± 4.38 ^ab^
Bacteroidetes	10.25 ± 2.54 ^ab^	16.41 ± 7.36 ^a–c^	24.60 ± 7.39 ^a–c^	18.01 ± 2.08 ^a–c^	35.88 ± 3.18 ^bc^	9.27 ± 8.18 ^ab^	19.14 ± 2.64 ^a–c^	11.13 ± 2.48 ^ab^	12.00 ± 7.81 ^a–c^	12.55 ± 9.05 ^a–c^	4.10 ± 0.25 ^a^	14.00 ± 0.92 ^a–c^	20.72 ± 12.52 ^a–c^	9.56 ± 0.27 ^ab^	39.21 ± 8.08 ^a–c^	18.32 ± 1.15 ^a–c^
Actinobacteria	0.42 ± 0.46 ^a^	0.09 ± 0.04 ^a^	0.31 ± 0.22 ^a^	0.07 ± 0.01 ^a^	0.88 ± 0.03 ^a^	0.41 ± 0.41 ^a^	0.06 ± 0.02 ^a^	0.07 ± 0.02 ^a^	0.22 ± 0.05 ^a^	1.00 ± 0.37 ^ab^	0.11 ± 0.01 ^a^	0.57 ± 0.30 ^ab^	0.30 ± 0.31 ^ab^	2.67 ± 0.43 ^b^	0.71 ± 0.74 ^a^	11.21 ± 0.90 ^c^
Verrucomicrobia	0.36 ± 0.09	5.27 ± 1.53	5.33 ± 6.54	4.97 ± 0.79	2.88 ± 3.87	0.31 ± 0.23	5.79 ± 3.72	9.42 ± 3.72	0.17 ± 0.11	2.82 ± 3.77	0.12 ± 0.02	0.97 ± 1.23	0.10 ± 0.01	0.07 ± 0.01	0.08 ± 0.01	4.71 ± 6.43

The values are presented as mean ± SD (n = 2 per group). Significant differences (*p* < 0.05) among animal groups, for each phylum, are indicated by different letters. CD: control animals following the control diet; OD: control animals following the oat flakes diet; CIC: control animals following the diet enriched with immobilized *L. rhamnosus* OLXAL-1 cells on oat flakes; CFC: control animals following the diet enriched with free *L. rhamnosus* OLXAL-1 cells; DCD: diabetic animals following the control diet; DOD: diabetic animals following the oat flakes diet; DIC: diabetic animals following the diet enriched with immobilized *L. rhamnosus* OLXAL-1 cells on oat flakes; DFC: diabetic animals following the diet enriched with free *L. rhamnosus* OLXAL-1 cells.

**Table 4:** Effect of the dietary intervention on fecal lactic acid and short-chain fatty acids (SCFAs) profile (μmol/g) at the beginning (week 0) and the end (week 4) of the 4-week protocol.

	CD		OD		CIC		CFC		DCD		DOD		DIC		DFC	
	Week 0	Week 4	Week 0	Week 4	Week 0	Week 4	Week 0	Week 4	Week 0	Week 4	Week 0	Week 4	Week 0	Week 4	Week 0	Week 4
Lactic acid	1.63 ± 0.91 ^a^	1.17 ± 0.52 ^a^	1.95 ± 0.33 ^a^	2.48 ± 1.10 ^a^	3.01 ± 0.65 ^a^	12.66 ± 2.51 ^b^	2.23 ± 1.13 ^a^	12.23 ± 0.60 ^b^	1.83 ± 0.53 ^a^	2.17 ± 1.30 ^a^	2.21 ± 0.79 ^a^	2.53 ± 0.66 ^a^	2.45 ± 0.91 ^a^	13.94 ± 3.89 ^b^	2.72 ± 0.91 ^a^	12.76 ± 2.52 ^b^
Acetic acid	19.11 ± 5.49 ^ac^	22.73 ± 10.30 ^a–c^	20.98 ± 3.09 ^a–c^	19.19 ± 8.22 ^a^	22.15 ± 5.49 ^a^	20.24 ± 5.86 ^a^	24.45 ± 3.89 ^a–d^	24.62 ± 3.13 ^a–d^	37.79 ± 4.93 ^b–e^	25.58 ± 9.69 ^a–d^	32.68 ± 5.35 ^a–e^	20.98 ± 6.97 ^ac^	38.8 ± 7.04 ^de^	47.83 ± 10.30 ^ef^	36.53 ± 6.59 ^b–e^	62.49 ± 8.32 ^f^
Propionic acid	1.77 ± 1.03	2.17 ± 1.72	1.87 ± 0.51	2.91 ± 1.94	1.96 ± 0.72	1.69 ± 1.80	1.08 ± 0.30	1.32 ± 0.61	3.51 ± 2.07	3.64 ± 2.80	2.11 ± 1.31	4.17 ± 2.53	1.37 ± 0.65	2.8 ± 1.00	2.30 ± 0.48	4.12 ± 2.48
Isobutyric acid	0.20 ± 0.11 ^a–c^	0.29 0.11 ^c^	0.13 ± 0.09 ^a–c^	0.10 ± 0.03 ^ab^	0.05 ± 0.03 ^a^	0.08 ± 0.03 ^ab^	0.15 ± 0.06 ^a–c^	0.17 ± 0.09 ^a–c^	0.11 ± 0.04 ^a–c^	0.24 ± 0.19 ^bc^	0.13 ± 0.06 ^a–c^	0.26 ± 0.06 ^bc^	0.06 ± 0.04 ^a^	0.05 ± 0.02 ^a^	0.09 ± 0.05 ^ab^	0.05 ± 0.05 ^a^
Butyric acid	1.71 ± 0.78 ^a^	1.76 ± 1.10 ^a^	1.69 ± 0.44 ^a^	1.11 ± 0.41 ^a^	1.42 ± 0.41 ^a^	4.84 ± 0.41 ^b^	1.20 ± 0.55 ^a^	1.49 ± 0.60 ^a^	0.95 ± 0.38 ^a^	1.55 ± 0.95 ^a^	1.05 ± 0.44 ^a^	1.79 ± 0.51 ^a^	1.35 ± 0.84 ^a^	1.18 ± 0.55 ^a^	1.46 ± 0.24 ^a^	1.22 ± 0.51 ^a^
Isovaleric acid	0.06 ± 0.01	0.15 ± 0.12	0.07 ± 0.05	0.12 ± 0.04	0.04 ± 0.02	0.14 ± 0.04	0.10 ± 0.06	0.13 ± 0.05	0.05 ± 0.02	0.12 ± 0.02	0.07 ± 0.04	0.10 ± 0.02	0.04 ± 0.02	0.10 ± 0.02	0.04 ± 0.05	0.10 ± 0.04
Valeric acid	0.23 ± 0.17	0.21 ± 0.17	0.15 ± 0.07	0.10 ± 0.07	0.07 ± 0.03	0.18 ± 0.10	0.14 ± 0.08	0.21 ± 0.10	0.09 ± 0.03	0.20 ± 0.03	0.10 ± 0.03	0.18 ± 0.07	0.07 ± 0.06	0.05 ± 0.03	0.10 ± 0.06	0.04 ± 0.02

The values are presented as mean ± SD (n = 6 per group). Significant differences (*p* < 0.05) among animal groups, for each fatty acid, are indicated by different letters. CD: control animals following the control diet; OD: control animals following the oat flakes diet; CIC: control animals following the diet enriched with immobilized *L. rhamnosus* OLXAL-1 cells on oat flakes; CFC: control animals following the diet enriched with free *L. rhamnosus* OLXAL-1 cells; DCD: diabetic animals following the control diet; DOD: diabetic animals following the oat flakes diet; DIC: diabetic animals following the diet enriched with immobilized *L. rhamnosus* OLXAL-1 cells on oat flakes; DFC: diabetic animals following the diet enriched with free *L. rhamnosus* OLXAL-1 cells.

## Data Availability

The data presented in this study are available on request from the corresponding author. The data are not publicly available due to restrictions of the funding authorities.

## References

[1] Furuyama K., Chera S., van Gurp L., Oropeza D., Ghila L., Damond N., Vethe H., Paulo J.A., Joosten A.M., Berney T. (2019). Diabetes relief in mice by glucose-sensing insulin-secreting human α-cells. Nature.

[2] Rawshani A., Landin-Olsson M., Svensson A.M., Nyström L., Arnqvist H.J., Bolinder J., Gudbjörnsdottir S. (2014). The incidence of diabetes among 0-34 year olds in Sweden: New data and better methods. Diabetologia.

[3] Diaz-Valencia P.A., Bougnères P., Valleron A.J. (2015). Global epidemiology of type 1 diabetes in young adults and adults: A systematic review. BMC Public Health.

[4] Atkinson M.A. (2012). The pathogenesis and natural history of type 1 diabetes. Cold Spring Harb. Perspect. Med..

[5] Niechciał E., Michalak M., Skowrońska B., Fichna P. (2024). Increasing trend of childhood type 1 diabetes incidence: 20-year observation from Greater Poland Province, Poland. Acta Diabetol..

[6] Wang Z., Xie Z., Lu Q., Chang C., Zhou Z. (2017). Beyond Genetics: What Causes Type 1 Diabetes. Clin. Rev. Allergy Immunol..

[7] Aschner P., Basit A., Fawwad A., Guariguata L., James S., Karuranga S., Malanda B., Mbanya J.C., O’neill S., Ogle G. (2022). IDF Atlas Reports. Int. Diabetes Fed..

[8] Mønsted M.Ø., Falck N.D., Pedersen K., Buschard K., Holm L.J., Haupt-Jorgensen M. (2021). Intestinal permeability in type 1 diabetes: An updated comprehensive overview. J. Autoimmun..

[9] Bielka W., Przezak A., Pawlik A. (2022). The role of the gut microbiota in the pathogenesis of diabetes. Int. J. Mol. Sci..

[10] Gradisteanu Pircalabioru G., Corcionivoschi N., Gundogdu O., Chifiriuc M.C., Marutescu L.G., Ispas B., Savu O. (2021). Dysbiosis in the development of type 1 diabetes and associated complications: From mechanisms to targeted gut microbes manipulation therapies. Int. J. Mol. Sci..

[11] Wen L., Ley R.E., Volchkov P.Y., Stranges P.B., Avanesyan L., Stonebraker A.C., Hu C., Wong F.S., Szot G.L., Bluestone J.A. (2008). Innate immunity and intestinal microbiota in the development of Type 1 diabetes. Nature.

[12] Giongo A., Gano K.A., Crabb D.B., Mukherjee N., Novelo L.L., Casella G., Drew J.C., Ilonen J., Knip M., Hyöty H. (2011). Toward defining the autoimmune microbiome for type 1 diabetes. ISME J..

[13] Beyan H., Wen L., Leslie R.D. (2012). Guts, germs, and meals: The origin of type 1 diabetes. Curr. Diab. Rep..

[14] Fuhri Snethlage C.M., Nieuwdorp M., Hanssen N.M.J. (2021). Faecal microbiota transplantation in endocrine diseases and obesity. Best Pract. Res. Clin. Endocrinol. Metab..

[15] Boerner B.P., Sarvetnick N.E. (2011). Type 1 diabetes: Role of intestinal microbiome in humans and mice. Ann. N. Y. Acad. Sci..

[16] Murri M., Leiva I., Gomez-Zumaquero J.M., Tinahones F.J., Cardona F., Soriguer F., Queipo-Ortuño M.I. (2013). Gut microbiota in children with type 1 diabetes differs from that in healthy children: A case-control study. BMC Med..

[17] Kin K.L., Lorca G.L., Gonzalez C.F. (2009). Biochemical properties of two cinnamoyl esterases purified from a *Lactobacillus johnsonii* strain isolated from stool samples of diabetes-resistant rats. Appl. Environ. Microbiol..

[18] Valladares R., Sankar D., Li N., Williams E., Lai K.K., Abdelgeliel A.S., Gonzalez C.F., Wasserfall C.H., Larkin J., Schatz D. (2010). *Lactobacillus johnsonii* N6.2 mitigates the development of type 1 diabetes in BB-DP rats. PLoS ONE.

[19] World Health Organization (WHO), Food and Agriculture Organization of the United Nations (FAO) (2002). Guidelines for the Evaluation of Probiotics in Food. Report of a Joint FAO/WHO Working Group on Drafting Guidelines for the Evaluation of Probiotics in Food.

[20] Ouwehand A.C. (2017). A review of dose-responses of probiotics in human studies. Benef. Microbes.

[21] Kwok K.O., Fries L.R., Silva-Zolezzi I., Thakkar S.K., Iroz A., Blanchard C. (2022). Effects of Probiotic Intervention on Markers of Inflammation and Health Outcomes in Women of Reproductive Age and Their Children. Front. Nutr..

[22] Plaza-Díaz J., Ruiz-Ojeda F.J., Vilchez-Padial L.M., Gil A. (2017). Evidence of the anti-inflammatory effects of probiotics and synbiotics in intestinal chronic diseases. Nutrients.

[23] Bezirtzoglou E., Stavropoulou E., Kantartzi K., Tsigalou C., Voidarou C., Mitropoulou G., Prapa I., Santarmaki V., Kompoura V., Yanni A.E. (2021). Maintaining digestive health in diabetes: The role of the gut microbiome and the challenge of functional foods. Microorganisms.

[24] Cristofori F., Dargenio V.N., Dargenio C., Miniello V.L., Barone M., Francavilla R. (2021). Anti-Inflammatory and Immunomodulatory Effects of Probiotics in Gut Inflammation: A Door to the Body. Front. Immunol..

[25] Dimitrellou D., Kandylis P., Sidira M., Koutinas A.A., Kourkoutas Y. (2014). Free and immobilized *Lactobacillus casei* ATCC 393 on whey protein as starter cultures for probiotic Feta-type cheese production. J. Dairy Sci..

[26] Nikolaou A., Galanis A., Kanellaki M., Tassou C., Akrida-Demertzi K., Kourkoutas Y. (2017). Assessment of free and immobilized kefir culture in simultaneous alcoholic and malolactic cider fermentations. LWT Food Sci. Technol..

[27] Nelios G., Prapa I., Nikolaou A., Mitropoulou G., Yanni A.E., Kostomitsopoulos N., Kourkoutas Y. (2023). Cereals and Fruits as Effective Delivery Vehicles of *Lacticaseibacillus rhamnosus* through Gastrointestinal Transit. Appl. Sci..

[28] Nelios G., Santarmaki V., Pavlatou C., Dimitrellou D., Kourkoutas Y. (2022). New Wild-Type *Lacticaseibacillus rhamnosus* Strains as Candidates to Manage Type 1 Diabetes. Microorganisms.

[29] Zhao Y., Li M., Wang Y., Geng R., Fang J., Liu Q., Kang S.G., Zeng W.C., Huang K., Tong T. (2023). Understanding the mechanism underlying the anti-diabetic effect of dietary component: A focus on gut microbiota. Crit. Rev. Food Sci. Nutr..

[30] Asnicar F., Berry S.E., Valdes A.M., Nguyen L.H., Piccinno G., Drew D.A., Leeming E., Gibson R., Le Roy C., Khatib H.A. (2021). Microbiome connections with host metabolism and habitual diet from 1,098 deeply phenotyped individuals. Nat. Med..

[31] Llanaj E., Dejanovic G.M., Valido E., Bano A., Gamba M., Kastrati L., Minder B., Stojic S., Voortman T., Marques-Vidal P. (2022). Effect of oat supplementation interventions on cardiovascular disease risk markers: A systematic review and meta-analysis of randomized controlled trials. Eur. J. Nutr..

[32] Kristek A., Schär M.Y., Soycan G., Alsharif S., Kuhnle G.G.C., Walton G., Spencer J.P.E. (2018). The gut microbiota and cardiovascular health benefits: A focus on wholegrain oats. Nutr. Bull..

[33] Thies F., Masson L.F., Boffetta P., Kris-Etherton P. (2014). Oats and CVD risk markers: A systematic literature review. Br. J. Nutr..

[34] Schuster J., Benincá G., Vitorazzi R., del Bosco S.M. (2015). Effects of oats on lipid profile, insulin resistance and weight loss. Nutr. Hosp..

[35] Bao L., Cai X., Xu M., Li Y. (2014). Effect of oat intake on glycaemic control and insulin sensitivity: A meta-analysis of randomised controlled trials. Br. J. Nutr..

[36] Paudel D., Dhungana B., Caffe M., Krishnan P. (2021). A review of health-beneficial properties of oats. Foods.

[37] Nikolaou A., Mitropoulou G., Nelios G., Kourkoutas Y. (2023). Novel Functional Grape Juices Fortified with Free or Immobilized *Lacticaseibacillus rhamnosus* OLXAL-1. Microorganisms.

[38] Furman B.L. (2021). Streptozotocin-Induced Diabetic Models in Mice and Rats. Curr. Protoc..

[39] Yanni A.E., Mitropoulou G., Prapa I., Agrogiannis G., Kostomitsopoulos N., Bezirtzoglou E., Kourkoutas Y., Karathanos V.T. (2020). Functional modulation of gut microbiota in diabetic rats following dietary intervention with pistachio nuts (*Pistacia vera* L.). Metab. Open.

[40] Kompoura V., Prapa I., Vasilakopoulou P.B., Mitropoulou G., Nelios G., Balafas E., Kostomitsopoulos N., Chiou A., Karathanos V.T., Bezirtzoglou E. (2023). Corinthian Currants Supplementation Restores Serum Polar Phenolic Compounds, Reduces IL-1beta, and Exerts Beneficial Effects on Gut Microbiota in the Streptozotocin-Induced Type-1 Diabetic Rat. Metabolites.

[41] Prapa I., Kompoura V., Pavlatou C., Nelios G., Mitropoulou G., Kostomitsopoulos N., Plessas S., Bezirtzoglou E., Karathanos V.T., Yanni A.E. (2024). Effects of Free or Immobilized Pediococcus acidilactici ORE5 on Corinthian Currants on Gut Microbiome of Streptozotocin-Induced Diabetic Rats. Microorganisms.

[42] Lagkouvardos I., Fischer S., Kumar N., Clavel T. (2017). Rhea: A transparent and modular R pipeline for microbial profiling based on 16S rRNA gene amplicons. PeerJ.

[43] Nikolaou A., Nelios G., Kanellaki M., Kourkoutas Y. (2020). Freeze-dried immobilized kefir culture in cider-making. J. Sci. Food Agric..

[44] Prapa I., Yanni A.E., Nikolaou A., Kostomitsopoulos N., Kalogeropoulos N., Bezirtzoglou E., Karathanos V.T., Kourkoutas Y. (2022). Dietary Pistachio (*Pistacia vera* L.) Beneficially Alters Fatty Acid Profiles in Streptozotocin-Induced Diabetic Rat. Appl. Sci..

[45] Al-Ishaq R.K., Abotaleb M., Kubatka P., Kajo K., Büsselberg D. (2019). Flavonoids and their anti-diabetic effects: Cellular mechanisms and effects to improve blood sugar levels. Biomolecules.

[46] Hossein-Nia B., Khorram S., Rezazadeh H., Safaiyan A., Ghiasi R., Tarighat-Esfanjani A. (2018). The effects of natural clinoptilolite and nano-sized clinoptilolite supplementation on lipid profile, food intakes and body weight in rats with streptozotocin-induced diabetes. Adv. Pharm. Bull..

[47] Jing S., Zhao Z., Wu J., Yan L.J. (2020). Antioxidative and hypoglycemic effect of ta-ermi extracts on streptozotocin-induced diabetes. Diabetes, Metab. Syndr. Obes..

[48] Zafar M., Naeem-ul-Hassan Naqvi S. (2010). Effects of STZ-Induced Diabetes on the Relative Weights of Kidney, Liver and Pancreas in Albino Rats: A Comparative Study. Int. J. Morphol..

[49] Wang R., Zhang Z., Aihemaitijiang S., Ye C., Halimulati M., Huang X., Qin H. (2022). Oat β Glucan Ameliorates Renal Function and Gut Microbiota in Diabetic Rats. Front. Nutr..

[50] Zhu Y., Dong L., Huang L., Shi Z., Dong J., Yao Y., Shen R. (2020). Effects of oat β-glucan, oat resistant starch, and the whole oat flour on insulin resistance, inflammation, and gut microbiota in high-fat-diet-induced type 2 diabetic rats. J. Funct. Foods.

[51] Arellano-García L., Macarulla M.T., Cuevas-Sierra A., Martínez J.A., Portillo M.P., Milton-Laskibar I. (2023). *Lactobacillus rhamnosus* GG administration partially prevents diet-induced insulin resistance in rats: A comparison with its heat-inactivated parabiotic. Food Funct..

[52] Nopparat J., Khuituan P., Peerakietkhajorn S., Teanpaisan R. (2023). Probiotics of *Lacticaseibacillus paracasei* SD1 and *Lacticaseibacillus rhamnosus* SD11 attenuate inflammation and β-cell death in streptozotocin-induced type 1 diabetic mice. PLoS ONE.

[53] Dikeman D.T., Westman E.C. (2021). Carbohydrate-restricted diets and Type 1 diabetes mellitus: Research considerations. Curr. Opin. Endocrinol. Diabetes Obes..

[54] Cano-Cano F., Gómez-Jaramillo L., Ramos-García P., Arroba A.I., Aguilar-Diosdado M. (2022). IL-1β Implications in Type 1 Diabetes Mellitus Progression: Systematic Review and Meta-Analysis. J. Clin. Med..

[55] Hajmrle C., Smith N., Spigelman A.F., Dai X., Senior L., Bautista A., Ferdaoussi M., MacDonald P.E. (2016). Interleukin-1 signaling contributes to acute islet compensation. JCI Insight.

[56] Fève B., Bastard J.P. (2009). The role of interleukins in insulin resistance and type 2 diabetes mellitus. Nat. Rev. Endocrinol..

[57] Peiró C., Lorenzo Ó., Carraro R., Sánchez-Ferrer C.F. (2017). IL-1β inhibition in cardiovascular complications associated to diabetes mellitus. Front. Pharmacol..

[58] Palmér R., Nyman E., Penney M., Marley A., Cedersund G., Agoram B. (2014). Effects of il-1β-blocking therapies in type 2 diabetes mellitus: A quantitative systems pharmacology modeling approach to explore underlying mechanisms. CPT Pharmacomet. Syst. Pharmacol..

[59] Aharon-Hananel G., Jörns A., Lenzen S., Raz I., Weksler-Zangen S. (2015). Antidiabetic effect of interleukin-1β antibody therapy through β-Cell protection in the cohen diabetes- sensitive rat. Diabetes.

[60] Spohn G., Schori C., Keller I., Sladko K., Sina C., Guler R., Schwarz K., Johansen P., Jennings G.T., Bachmann M.F. (2014). Preclinical efficacy and safety of an anti-IL-1β vaccine for the treatment of type 2 diabetes. Mol. Ther. Methods Clin. Dev..

[61] Meydani M. (2009). Potential health benefits of avenanthramides of oats. Nutr. Rev..

[62] Suchecka D., Błaszczyk K., Harasym J., Gudej S., Wilczak J., Gromadzka-ostrowska J. (2017). Impact of purified oat 1-3, 1-4- b -d-glucan of different molecular weight on alleviation of inflammation parameters during gastritis. J. Funct. Foods.

[63] Koenig R., Dickman J.R., Kang C., Zhang T., Chu Y.F., Ji L.L. (2014). Avenanthramide supplementation attenuates exercise-induced inflammation in postmenopausal women. Nutr. J..

[64] Wang Y., Qi W., Guo X., Song G., Pang S., Fang W., Peng Z. (2022). Effects of oats, tartary buckwheat, and foxtail millet supplementation on lipid metabolism, oxido-inflammatory responses, gut microbiota, and colonic SCFA composition in high-fat diet fed rats. Nutrients.

[65] Zheng D., Dou J., Liu G., Pan Y., Yan Y., Liu F., Gaisano H.Y., Lu J., He Y. (2019). Association between triglyceride level and glycemic control among insulin-treated patients with type 2 diabetes. J. Clin. Endocrinol. Metab..

[66] Guo R., Wei L., Cao Y., Zhao W. (2024). Normal triglyceride concentration and the risk of diabetes mellitus type 2 in the general population of China. Front. Endocrinol..

[67] Zhao J., Zhang Y., Wei F., Song J., Cao Z., Chen C., Zhang K., Feng S., Wang Y., Li W.D. (2019). Triglyceride is an independent predictor of type 2 diabetes among middle-aged and older adults: A prospective study with 8-year follow-ups in two cohorts. J. Transl. Med..

[68] Guy J., Ogden L., Wadwa R.P., Hamman R.F., Mayer-Davis E.J., LIese A.D., D’Agostino R., Marcovina S., Dabelea D. (2009). Lipid and lipoprotein profiles in youth with and without type 1 diabetes: The SEARCH for diabetes in youth case-control study. Diabetes Care.

[69] Unger G., Benozzi S.F., Perruzza F., Pennacchiotti G.L. (2014). Triglycerides and glucose index: A useful indicator of insulin resistance. Endocrinol. Nutr. (Engl. Ed.).

[70] Wunderling K., Zurkovic J., Zink F., Kuerschner L., Thiele C. (2023). Triglyceride cycling enables modification of stored fatty acids. Nat. Metab..

[71] Yan G., Li S., Wen Y., Luo Y., Huang J., Chen B., Lv S., Chen L., He L., He M. (2022). Characteristics of intestinal microbiota in C57BL/6 mice with non-alcoholic fatty liver induced by high-fat diet. Front. Microbiol..

[72] Ma Y., Deng X., Yang X., Wang J., Li T., Hua G., Han D., Da L., Li R., Rong W. (2022). Characteristics of Bacterial Microbiota in Different Intestinal Segments of Aohan Fine-Wool Sheep. Front. Microbiol..

[73] Leeming E.R., Johnson A.J., Spector T.D., Roy C.I.L. (2019). Effect of diet on the gut microbiota: Rethinking intervention duration. Nutrients.

[74] Edith Marius F.K., François Z.N., Pierre Marie K., Rui Yan W., Taicheng Z., Li Y. (2018). Screening and Characterization of *Lactobacillus* sp. from the Water of Cassava’s Fermentation for Selection as Probiotics. Food Biotechnol..

[75] Liu P., Wang Y., Yang G., Zhang Q., Meng L., Xin Y., Jiang X. (2021). The role of short-chain fatty acids in intestinal barrier function, inflammation, oxidative stress, and colonic carcinogenesis. Pharmacol. Res..

[76] Wang G., Si Q., Yang S., Jiao T., Zhu H., Tian P., Wang L., Li X., Gong L., Zhao J. (2020). Lactic acid bacteria reduce diabetes symptoms in mice by alleviating gut microbiota dysbiosis and inflammation in different manners. Food Funct..

[77] Pahwa R., Balderas M., Jialal I., Chen X., Luna R.A., Devaraj S. (2017). Gut Microbiome and Inflammation: A Study of Diabetic Inflammasome-Knockout Mice. J. Diabetes Res..

[78] Demirci M., Bahar Tokman H., Taner Z., Keskin F.E., Çağatay P., Ozturk Bakar Y., Özyazar M., Kiraz N., Kocazeybek B.S. (2020). Bacteroidetes and Firmicutes levels in gut microbiota and effects of hosts TLR2/TLR4 gene expression levels in adult type 1 diabetes patients in Istanbul, Turkey. J. Diabetes Complicat..

[79] Menofy N.G.E., Radwan H.M., Radwan S.M.R. (2020). The Diversity of Gut Microbiota among Type 1 and Type 2 Egyptian Diabetic Patients. Egypt. J. Med. Microbiol..

[80] Lippert K., Kedenko L., Antonielli L., Kedenko I., Gemeier C., Leitner M., Kautzky-Willer A., Paulweber B., Hackl E. (2017). Gut microbiota dysbiosis associated with glucose metabolism disorders and the metabolic syndrome in older adults. Benef. Microbes.

[81] Turnbaugh P.J., Hamady M., Yatsunenko T., Cantarel B.L., Duncan A., Ley R.E., Sogin M.L., Jones W.J., Roe B.A., Affourtit J.P. (2009). A core gut microbiome in obese and lean twins. Nature.

[82] Wang Y., Dilidaxi D., Wu Y., Sailike J., Sun X., Nabi X. (2020). hua Composite probiotics alleviate type 2 diabetes by regulating intestinal microbiota and inducing GLP-1 secretion in db/db mice. Biomed. Pharmacother..

[83] Hänninen A., Toivonen R., Pöysti S., Belzer C., Plovier H., Ouwerkerk J.P., Emani R., Cani P.D., De Vos W.M. (2018). *Akkermansia muciniphila* induces gut microbiota remodelling and controls islet autoimmunity in NOD mice. Gut.

[84] Peng L., Li Z.R., Green R.S., Holzman I.R., Lin J. (2009). Butyrate enhances the intestinal barrier by facilitating tight junction assembly via activation of AMP-activated protein kinase in Caco-2 cell monolayers. J. Nutr..

[85] Mandaliya D.K., Seshadri S. (2019). Short Chain Fatty Acids, pancreatic dysfunction and type 2 diabetes. Pancreatology.

[86] Maslowski K.M., Vieira A.T., Ng A., Kranich J., Sierro F., Di Y., Schilter H.C., Rolph M.S., MacKay F., Artis D. (2009). Regulation of inflammatory responses by gut microbiota and chemoattractant receptor GPR43. Nature.

[87] Wang C., Wang X., Huang Y., Bu X., Xiao S., Qin C., Qiao F., Qin J.G., Chen L. (2020). Effects of dietary T-2 toxin on gut health and gut microbiota composition of the juvenile Chinese mitten crab (*Eriocheir sinensis*). Fish Shellfish Immunol..

[88] Liu Y.X., Qin Y., Chen T., Lu M., Qian X., Guo X., Bai Y. (2021). A practical guide to amplicon and metagenomic analysis of microbiome data. Protein Cell.

[89] Corrêa R.O., Vieira A., Sernaglia E.M., Lancellotti M., Vieira A.T., Avila-Campos M.J., Rodrigues H.G., Vinolo M.A.R. (2017). Bacterial short-chain fatty acid metabolites modulate the inflammatory response against infectious bacteria. Cell. Microbiol..

[90] Frost G., Sleeth M.L., Sahuri-Arisoylu M., Lizarbe B., Cerdan S., Brody L., Anastasovska J., Ghourab S., Hankir M., Zhang S. (2014). The short-chain fatty acid acetate reduces appetite via a central homeostatic mechanism. Nat. Commun..

[91] Gao Z., Yin J., Zhang J., Ward R.E., Martin R.J., Lefevre M., Cefalu W.T., Ye J. (2009). Butyrate improves insulin sensitivity and increases energy expenditure in mice. Diabetes.

[92] Traisaeng S., Batsukh A., Chuang T.H., Herr D.R., Huang Y.F., Chimeddorj B., Huang C.M. (2020). *Leuconostoc mesenteroides* fermentation produces butyric acid and mediates Ffar2 to regulate blood glucose and insulin in type 1 diabetic mice. Sci. Rep..

[93] Plummer E.L., Bradshaw C.S., Doyle M., Fairley C.K., Murray G.L., Bateson D., Masson L., Slifirski J., Tachedjian G., Vodstrcil L.A. (2021). Lactic acid-containing products for bacterial vaginosis and their impact on the vaginal microbiota: A systematic review. PLoS ONE.

[94] Singh R., Gholipourmalekabadi M., Shafikhani S.H. (2024). Animal models for type 1 and type 2 diabetes: Advantages and limitations. Front. Endocrinol..

[95] Prapa I., Yanni A.E., Kompoura V., Mitropoulou G., Panas P., Kostomitsopoulos N., Kourkoutas Y. (2024). Functional Modulation of Gut Microbiota and Blood Parameters in Diabetic Rats Following Dietary Intervention with Free or Immobilized *Pediococcus acidilactici* SK Cells on Pistachio Nuts. Nutrients.

[96] Zhu W., Zhang X., Wang D., Yao Q., Ma G.-L., Fan X. (2024). Simulator of the Human Intestinal Microbial Ecosystem (SHIME^®^): Current Developments, Applications, and Future Prospects. Pharmaceuticals.

[97] Liu T., Zhang L., Joo D., Sun S.C. (2017). NF-κB signaling in inflammation. Sig. Transduct. Target. Ther..

[98] Adamska E., Ostrowska L., Goŕska M., Kreţowski A. (2014). The role of gastrointestinal hormones in the pathogenesis of obesity and type 2 diabetes. Prz. Gastroenterol..

[99] Fan S., Xu Y., Lu Y., Jiang Z., Li H., Morrill J.C., Cai J., Wu Q., Xu Y., Xue M. (2021). A neural basis for brain leptin action on reducing type 1 diabetic hyperglycemia. Nat. Commun..

